# Clinical Features and Short-Term Outcomes of Bariatric Surgery in Morbidly Obese Patients: Institutional Experience at a Rural Hospital

**DOI:** 10.1089/bari.2020.0110

**Published:** 2021-03-15

**Authors:** Kazim Senol, Murat Ferhat Ferhatoglu, Aysen Akkurt Kocaeli, Halit Ziya Dundar, Ekrem Kaya

**Affiliations:** ^1^ Department of General Surgery, Faculty of Medicine, Uludag University, Bursa, Turkey.; ^2^ Department of General Surgery, Faculty of Medicine, Okan University, İstanbul, Turkey.; ^3^ Department of Endocrinology, Bursa State Hospital, Bursa, Turkey.

**Keywords:** obesity, rural surgery, comorbidity resolution

## Abstract

***Objective:*** To prospectively evaluate the postoperative morbidity, mortality, and weight loss evolution of patients who underwent a bariatric procedure during 1 year of follow-up.

***Methods:*** Since July 2016, a total of 101 patients' data have been prospectively registered in a database. Comorbidities, operating time, hospital stay, early and late complications rate, and weight loss evolution after 1 year of follow-up were recorded.

***Results:*** The mean age was 38.41 ± 11.05 years with a mean body mass index (BMI) of 49.02 ± 5.89 kg/m^2^ (range 38–67). Laparoscopic sleeve gastrectomy (LSG) was performed in 93 patients (92.07%) and Roux-en-Y gastric bypass (RNYGB) in 8 patients (7.92%). Thirty-day morbidity rate was 7.92% (8/101). Within a mean 9.32 ± 2.25 (range 1–19) months follow-up time, mean percent of the excess of weight loss of 1st, 6th, and 12th months were 22.7 ± 6.1, 67.2 ± 11.2, and 81.4 ± 10.5, respectively. Diabetes (*n* = 38, 37.6%), hypertension (*n* = 13, 12.9%), and obstructive sleep apnea (*n* = 5, 4.9%) were resolved in 76%, 68.4%, and 100% of the patients, respectively (*p* < 0.001).

***Conclusions:*** LSG and RNYGB are safe and highly effective, particularly in patients with a BMI >50 kg/m^2^. Both techniques have been presented with better clinical outcomes regarding significant comorbidity resolution in the early evolution of weight loss.

## Introduction

Obesity is an actual health problem globally and increases the risk for several cardiometabolic diseases, including diabetes, hypertension, dyslipidemia, and coronary heart diseases. World Health Organization (WHO) and National Institutes of Health (NIH) have classified obesity with weight status based on body mass index (BMI).^1,2^ Epidemiological studies have demonstrated that an increase in BMI rates is strongly associated with severe medical conditions and elevated mortality rates.^3^ Medical therapy, diet, and lifestyle modifications have slight effects on weight loss and improvement in obesity-related comorbidities.^4^ Therefore, bariatric surgery has been propagated with better clinical outcomes in managing obesity and obesity-related health problems.

Bariatric surgery provides a significant decrease in BMI in accordance with satisfactory resolution rates in diabetes, hypertension, and dyslipidemia during both short- and long-term follow-up.^5–7^ In 1991, NIH Consensus Development Panel indicated a stratified nonsurgical therapy in the management of obesity; including dietary regimen, appropriate exercise, behavior modification and psychological support, and surgical treatment, in patients with BMI >40 kg/m^2^ and BMI >35 kg/m^2^ with at least two associated comorbidities.^8^ As indicated in the guidelines, professionals should arrange each treatment recommendation, and patients should receive an appropriate medical approach regarding comorbid diseases. A multidisciplinary approach to medical, surgical, psychiatric, and nutritional support was considered an integrated lifelong medical surveillance after surgery.

Patients living in rural areas have limited access to qualified health care services.^9^ Obese patients also lack appropriate medical and surgical care concerning obesity and obesity-related diseases in urbanized districts. Rural surgeons usually perform diverse case mix including thyroidectomies, laparotomies, emergency procedures, screening endoscopies, and laparoscopic procedures such as cholecystectomy, hysterectomy, and herniorrhaphy, in the daily routine practice. However, new trends and advancements in medical technology have presented several challenges in surgical specialties. Therefore, surgeons practicing in the rural setting seek advanced surgical procedures, including bariatric surgery, to provide the opportunity to specialize and to gain experience.^10^

This study was aimed to prospectively evaluate the short-term postoperative morbidity, mortality, and weight loss evolution of obese patients with several comorbid diseases who underwent a bariatric procedure under a review of a multidisciplinary team in a rural setting.

## Materials and Methods

### Patient population and preoperative evaluation

A total of 101 patients who underwent bariatric surgery by the same surgical team from July 2016 to December 2017 were enrolled in this observational cohort study. The study was approved and reviewed by the institutional ethics committee under the American Association for Clinical Endocrinologists, The Obesity Society, and The American Society for Metabolic and Bariatric Surgery guidelines, and the provision of the Declaration of Helsinki (01.20/117). All patients provided written informed consent to participate in the study, and additional informed consent was obtained before any surgical procedure. All patients were evaluated preoperatively by endocrinology, psychology, pulmonary, and anesthesiology specialists. Complete blood count tests, liver, thyroid, renal, and pulmonary function tests with upper gastrointestinal endoscopy and upper abdominal ultrasonography were routinely performed in all patients. Adrenal functions were evaluated with plasma cortisol levels and a 1 mg dexamethasone suppression test. Patients were reviewed by a multidisciplinary team, including a diabetologist, a dietitian, and a nurse with regular follow-up at baseline and 1, 3, 6, and 12 months after the study entry.

### Surgical technique

All patients were given clear liquid diets before 2 weeks of surgery. Preoperative subcutaneous low molecular weight heparin prophylaxis and second-generation cephalosporins (2 g <120 kg, 3 g >120 kg) were applied routinely. Urinary catheter, anti-emboli socks, and intermittent pneumatic compressing devices were used in the operating room preoperatively.

The patient was placed supine with a slight leg split position in which the operating surgeon was positioned between the legs and one assistant on the left side of the patient to hold the scope and assist the tissue retraction. Display screens were positioned to the right side, and head of the patient for a better view. Veress needle was routinely inserted to create the pneumoperitoneum to set the intra-abdominal pressure with CO_2_ gas insufflation at ∼13 mmHg. After achieving the adequate pneumoperitoneum, a total of five trocars were inserted; 10 or 12 mm trocar in the upper abdomen 1–2 cm above the umbilicus for the scope, 12 or 15 mm trocars in the right and left upper quadrants for dissection and stapling and 5 mm trocar to the subxiphoid for liver retraction and left anterior axillary line for omental tissue and gastric retraction to assist the surgeon as well. A subxiphoid trocar was then removed to place Nathanson liver retractor to get better exposure around the gastroesophageal junction. The patient was positioned to the reverse Trendelenburg, and the pylorus was identified. The anesthesiologist utilized a 36F calibration tube, and stomach ingredients were aspirated before dissecting the greater curvature.

The first step to perform the laparoscopic sleeve gastrectomy (LSG) was to criticize two landmarks by liberating the omentum from greater curvature by dividing gastrocolic and gastrosplenic ligaments with Ligasure^™^. The distal landmark of the greater curvature dissection was determined 4 cm proximal to the pylorus to preserve the excreting function of the antrum. The proximal landmark of the dissection was aimed to the left crus of the diaphragm to avoid inadequate dissection, which causes further weight regain and also to preserve blood supply for possible staple line leakage. The fat pad on the gastroesophageal junction was also dissected and removed before transection of the stomach. Following the great curvature's liberation from the omentum, the stomach was divided from the antrum toward the angle of his by using the Echelon Flex^™^ Lineer Cutter, 60 mm loaded with ECR60 cartridges (Ethicon Endo-Surgery). Initial stapling of the gastric antrum was essential because of its wall thickness and was performed with a green cartridge (closed heights 2.0 mm, 4.1/60 mm) followed by with sequential gold cartridge for corpus and fundus (closed heights 1.5 mm, 3.5/60 mm).

The first step to perform the Roux-en-Y gastric bypass (RNYGB) was to dissect the left diaphragmatic pillar with Ligasure to prepare the gastric pouch. Dissection of the omentum was essential to identify lesser sac, retrogastric space, and angle of His. The fat pad on the anterior surface of the stomach should also be removed. The stomach was transected through this window with Echelon Flex Lineer Cutter, 60 mm loaded with ECR60 gold cartridges between two landmarks 3–4 cm below the esophagogastric junction on the left side and angle of His on the right side to create a 30–40 cm^3^ gastric pouch. The second step was to create a jejunal loop, the Roux limb of the RNYGB procedure, to perform the gastrojejunal anastomosis. Treitz was identified, and small intestines were measured with the help of the graspers up to the terminal ileum. The jejunal segment 50 cm below the treitz was marked with a polyglactin Vicryl^™^ suture. The length of the biliopancreatic limb and common limb should be discussed in each patient individually, considering the comorbid diseases such as diabetes mellitus. Patients with diabetes and super-obese patients should be considered for an anastomosis ∼100 cm distal to the gastrojejunostomy. A seromucosal full-thickness cut was performed with the Ligasure at the anterior surface of the gastric pouch and marked proximal jejunal segment to create a window for stapling device. An Echelon Flex Lineer Cutter, 60 mm loaded with ECR60 gold cartridge, was inserted to the abdomen from the left upper 12 mm trocar, and the jejunal loop was mobilized to the gastric pouch. A 2 cm in diameter antecolic isoperistaltic gastrojejunal anastomosis was performed with the stapling device. The gastric tube was inserted through the anastomosis to calibrate the anastomosis, and the openings and the staple line were sutured continuously in two layers with V-Lock 3.0^™^. The Roux limb of the procedure was sutured to the remnant stomach with Ethibond 2-0^™^ sutures to avoid further twist of the anastomosis. The third step was the anastomosis of the pancreatobiliary limb to the jejunum distal to the gastrojejunostomy anastomosis. The jejunum was measured 100 cm distal to the gastrojejunostomy and mobilized close to the pancreatobiliary limb. A seromucosal full-thickness cut was performed with the Ligasure at the anterior surfaces of the jejunal segments. An Echelon Flex Lineer Cutter, 60 mm loaded with ECR60 gold cartridge, was inserted to the abdomen from the left upper 12 mm trocar, and isoperistaltic side-to-side jejunojejunostomy was performed with the stapling device. The openings of the jejunojejunostomy anastomosis were sutured continuously in one layer with V-Lock 3.0. The last step was achieved with the stapling device by transecting the jejunum between gastrojejunostomy and jejunojejunostomy loops. The Petersen mesenteric window was sutured with nonabsorbable 2-0 silk sutures continuously.

To reduce the risks of staple line complications, compression for 2 min before firing should be taken into account in both LSG and RNYGB procedures. Staple line reinforcement was achieved in all patients with V-Lock seroserous, running, imbricating sutures, and fibrin sealant material (Tisseel^™^; Baxter, Deerfield, IL) consecutively. The methylene blue injection from the calibration tube was routinely used to test for the leakage after placing white sponges through the staple line. The resected specimen was extracted from the abdominal cavity through a 12 mm trocar, which was placed lateral to the optical trocar in the left quadrant. A 7 mm suction drain was placed laterally to the staple line from the left quadrant 5 mm trocar site. The fascia of the 12 mm port openings was closed with 2/0 Vicryl sutures.

### Postoperative follow-up

All patients were followed-up in the intensive care unit postoperatively for close cardiac and renal monitoring. Antibiotic prophylaxis was ordered for 24 h. Early mobilization on the postoperative 6th hour was achieved. On the postoperative first day, all patients were transferred into the surgery department. Esophagogastric barium swallow test was performed on the postoperative second day, and patients with negative leakage test were given a clear liquid diet. Patients who tolerated oral intake ambulated independently and were relieved from pain with light analgesics were discharged on the postoperative fourth or fifth day. The drain was removed before discharge. Anticoagulant prophylaxis and soft diet were maintained for 30 days after surgery.

### Statistical analysis

Demographic and categorical data were expressed as absolute or as frequencies. Continuous variables were presented as mean value and standard deviation. BMI, BMI loss percent, excess weight loss (EWL), and EWL percent were calculated. Mann–Whitney *U* test and chi-squared test were used to assess differences when appropriate. Patients were grouped according to BMI, and the difference between groups was calculated with Kruskal–Wallis and one-way analysis of variance tests. A value of *p* < 0.05 was considered as tatistically significant. The statistical analyses were performed with SPSS Version 20.0 (Chicago, IL).

## Results

A total of 101 patients were included, 59.4% of whom were women. The mean age was 38.41 ± 11.05 years and the mean BMI was 49.02 ± 5.89 kg/m^2^ (range 38–67). Eleven patients (10.8%) presented a BMI <40 kg/m^2^. LSG was performed in 93 patients (92.07%) and RNYGB in 8 patients (7.92%). Cholecystectomy and hiatal hernia repair were performed in 12 (11.88%) and 10 (9.9%) patients, respectively. Conversion to open surgery was not necessary.

Patients' clinical and demographic variables are given in Tables 1 and 2.

**Table 1.** Clinical and Demographic Variables of the Patients

Variables	Sleeve gastrectomy group (*n* = 93)	Bypass group (*n* = 8)	*p*
Age, years, mean	35.89 ± 9.35	40.38 ± 8.65	0.172
Gender, female/male	54/39	6/2	0.584
Length, cm, mean	165.08 ± 8.28	161.13 ± 12.10	0.138
Weight, kg, mean	128.06 ± 20.19	133.88 ± 24.70	0.567
BMI, kg/m^2^, mean	47.12 ± 6.32 kg/m^2^ (range 35–65)	51.17 ± 4.77 kg/m^2^ (range 42–56)	0.039
BMI <40 kg/m^2^	11 (10.8%)	—	0.025
BMI 40–50 kg/m^2^	59 (58.4%)	3 (3%)	
BMI >50 kg/m^2^	23 (22.8%)	5 (5%)	
Excess weight, kg, mean	69.42 ± 16.05 (47–115)	65.50 ± 14.41 (55–82)	0.582
Operation time, min, mean	74 ± 12.5 (65–127)	125 ± 21.3 (102–155)	0.052
Hospital stay, days	3.8 ± 1.3 (1–19)	5.1 ± 1.7 (4–17)	0.048
Follow-up time, months, mean	9.3 ± 2.2 (1–17)		

BMI, body mass index.

**Table 2.** Pre- and Postoperative Comorbidity and Mortality of the Patients

	Sleeve gastrectomy group (*n* = 93)	Bypass group (*n* = 8)
Conversion to open surgery	None	None
Mortality	None	None
Reoperation	2 (1.98%)	—
Stapler line leakage	4 (3.96%)	1 (0.99)
Omental bleeding	1 (0.9%)	—
Pulmonary embolism	1 (0.9%)	—

### Morbidity and mortality

The mortality rate was 0%. The leakage rate was 4.95% (*n* = 5) and, the total 30-day morbidity rate was 7.92% (8/101). The mean hospital stay was 3.5 ± 0.7 (1–19) days. Perioperative stapler line complications were successfully managed in two LSG patients with stapler line reinforcement techniques. Stapler line leakage in one LSG case and one RNYGB case that occurred in early follow-up on the postoperative second day, were proved with swallow test, and treated with intragastric stenting through the conservative approach. One patient with LSG was operated on the fifth postoperative day owing to initial methylene-blue leakage into the drain consequent to findings of abdominal discomfort, and the leakage line was one-layer sutured. The postoperative hemoperitoneum because of omental hemorrhage was drained by percutaneous intervention in one LSG patient. One patient with LSG presented with tachypnea, chest pain, and normal chest computed tomography angiography findings were evaluated as suspicious for pulmonary embolism and treated with therapeutic anticoagulant doses of low-molecular-weight heparinization and broncodilator treatment as well.

### Weight loss

**FIG. 1.**
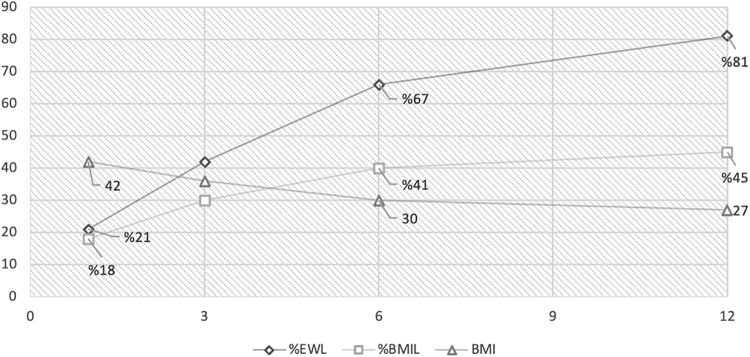
Body mass index, percentage of body mass index and excess weight loss of the patients during 1-year follow-up.

With a mean follow-up 9.32 ± 2.25 (range 1–19) months, the mean percent of the excess weight loss (% EWL) was 22.7 ± 6.1 at the first month, 43.1 ± 9.8 at the third month, 67.2 ± 11.2 at the sixth month, 81.4 ± 10.5 at first year. The mean percent of the excess BMI loss (% EBMIL) was 17.9 ± 5.2 in the first month, 31.3 ± 5.2 in the third month, 41.2 ± 4.7 at the sixth month, and 45.4 ± 4.8 at first year. Patients with preoperative BMI >50 kg/m^2^ achieved greater weight loss and increased rates of total body mass index loss % and % EWL than did the overall study population (*p* < 0.05). Weight loss status during a 1-year follow-up in all patients and subgroup analyses of patients according to BMI are given in Figure 1 and Tables 3 and 4.

**Table 3.** Weight Loss Evolution of the Patients

	Preoperative	First month	Third month	Sixth month	First year
Patients	101	101	89	75	70
BMI, kg/m^2^, mean	49.1 ± 5.9	42.4 ± 5.8	36.6 ± 5.2	30.4 ± 4.5	27.3 ± 4.6
% BMIL, %, mean	—	17.9 ± 5.2	31.3 ± 5.2	41.2 ± 4.7	45.4 ± 4.8
EWL, kg, mean	76.1 ± 16.5	22.1 ± 6.3	44.3 ± 10.2	66.4 ± 10.6	81.3 ± 10.5
% EWL, %, mean	—	22.7 ± 6.1	43.1 ± 9.8	67.2 ± 11.2	81.4 ± 10.5
TWL, kg, mean	—	16.3 ± 5.3	31.1 ± 8.8	47.2 ± 10.8	59.2 ± 18.3

BMI, body mass index; BMIL, body mass index loss; EWL, excess weight loss; TWL, total weight loss.

**Table 4.** Subgroup Analyses and Weight Loss Evolution of Patients According to Preoperative Body Mass Index

Patients (*N* = 101)	% TBMIL	% EWL	*p*
BMI <40 kg/m^2^	31.6 ± 2.8	71.1 ± 10.4	<0.001
BMI 40–50 kg/m^2^	39.4 ± 3.8	64.3 ± 8.3	
BMI >50 kg/m^2^	44.1 ± 5	85.3 ± 11.6	

TBMIL, total body mass index loss.

### Resolution of comorbidities

Fifty-seven (56.4%) patients presented with at least one comorbid disease. Thirty-eight (37.6%) of them were prescribed regular medication for hypertension and diabetes regulation. Mean HbA1c levels in diabetic patients on admission was 7.74 ± 1.52, and were 6.44 ± 0.63, 5.78 ± 0.99, and 5.18 ± 0.68 on the 3rd, 6th, and 12th months, respectively. Antidiabetic drug use was reduced with regulated fasting blood glucose levels and HbA1c levels in diabetic patients (*n* = 38, 37.6%) during endocrinology consultations, thus interpreted as diabetes resolution in 76% of the patients in the first year. HbA1c levels were decreased in all subgroups of obese patients but were found to be insignificant in between groups (*p* > 0.05). In addition, improvement in hypertension was achieved in 10 of 13 patients (12.9%) with a 68.4% reduction in antihypertensive medication history. All patients with obstructive sleep apnea syndrome (*n* = 5, 4.95%) were relieved from airway occlusion episodes and nasal continuous positive airway pressure support during sleep in the first year after surgery. Other obesity-related comorbidities, including joint and musculoskeletal pain, fibromyalgia, hyperlipidemia, lower extremity edema, sleep disorders, fatigue, and exhaustion, were also resolved with excessive weight loss during follow-up. Obesity-related comorbidities, prevalence rates before surgery, and postoperative resolution rates are given in Table 5.

**Table 5.** Remission Rates of Comorbidities

Comorbidities	Patients (*N* = 101)	Resolution
Diabetes mellitus, %	38 (37.6%)	29 (76.3%)
Hypertension, %	19 (18.8%)	13 (68.4%)
OSAS, %	5 (4.95%)	5 (100%)
PCOS, %	2 (1.98%)	1 (50%)
Others	14 (13.86%)	10 (71.4%)

OSAS, obstructive sleep apnea syndrome; PCOS, polycystic ovary syndrome.

## Discussion

Bariatric surgery is considered the most effective treatment modality for morbid obesity, whereas nonsurgical therapy is associated with 5–8% of weight loss and improvement in comorbid diseases.^3^ RNYGB and LSG come into prominence as feasible and safe techniques in the clinical setting. American Society for Metabolic and Bariatric Surgery has recently mentioned that LSG is the most preferred procedure among bariatric surgeons since 2013.^11^ LSG is also considered a more straightforward procedure with a short learning curve and has surgical superiority according to the RNYGB with lower early postoperative complication rates.^12–14^ Although emerging trends in bariatric surgery put forward LSG as the first-line treatment for morbid obesity among surgeons because of these advantages,^15^ life-threatening complications have been demonstrated in both techniques.

Overall postoperative complication rates of LSG and RNYGB vary between 8.4–13.2%^16^ and 10–27.4%,^17^ respectively. The complications are considered in the early and late period of the postoperative follow-up. The majority of the complications are demonstrated during the late period and include; gastroesophageal reflux (23%), vomiting (18%), stricture (2%), stenosis (3%), incisional hernia, and gastrocutaneous fistula. Early complications such as proximal leakage and bleeding from the staple line are rare but may worsen the clinical course. Among all complications, staple line leakage and bleeding are defined as the most serious complications with lower incidence rates of 0.5% and 0.27% in LSG, respectively.^6^

Unfortunately, recent reports have demonstrated increased leakage rates for RNYGB with 0–8.3% compared with LSG.^18^ In our study, early postoperative complication rates were similar to the literature but increased peri- and postoperative stapler line leakage rates were noteworthy, especially in the first 30 cases. Regarding perioperative complications and reoperation rates, the learning curve has been mentioned as an essential factor in several studies by identifying intraoperative difficulties, the number of stapler firings, adverse effects, and operative time.^19,20^ It has been argued that bariatric surgeons should perform adequate amounts of procedures to reach proficiency in bariatric procedures. Early experience in the learning curve resulted in a significant decrease in operative time and hospital stay as opposed to postoperative mortality, morbidity, and conversion rates.^19^ However, such independent risk factors associated with patient-specific comorbidities have been documented in the management and postoperative outcomes of LSG regardless of surgical experience and proficiency. Patients older than 65 years with a history of diabetes and hypertension and prior treatment with anticoagulants were determined as nonmodifiable risk factors.^21^ Subsequent to the first 30 cases, peri- or postoperative complications such as staple line leakage and bleeding should be avoided if the experience of the operation team has been improved while the learning curve was achieved. Besides that, optimization of surgical technique and operative management, advancement in surgical skills, and utilization of staple line reinforcement techniques resulted in favorable outcomes and reduced the complication rates to almost 0% in this study.

Randomized and nonrandomized clinical trials in bariatric surgery presented improvement in obesity and obesity-related comorbidities. Swiss Multicentre Bypass or Sleeve Study (SM-BOSS) among four bariatric centers by Peterli *et al.* have demonstrated a favorable increase in EBMIL and resolution of comorbidities with an enhanced quality of life between baseline, first and fifth postoperative years in both LSG and RNYGB with an insignificant difference between groups.^12,22^ In this study, similar findings were noticed in all BMI subgroups regardless of the surgical approach, but patients with BMI >50 kg/m^2^ achieved better weight loss and % EWL during first-year follow-up with a significant difference (*p* < 0.001). Helmio *et al.* have presented in a randomized controlled multicenter study comparing short-term outcomes of LSG and RNYGB that there was no statistically significant difference in weight loss, resolution of obesity-related comorbidities, and complications.^23^ Type 2 diabetes and hypertension were resolved in RNYGB and LSG groups during short-term follow-up as 93.3% versus 84.3% (*p* = 0.58) and 81.9% versus 76.8% (*p* = 0.70), respectively. However, this study's long-term outcomes revealed that RNYGB was associated with increased rates of EWL and a statistically significant improvement in hypertension at 5 years compared with LSG.^24^ Concerning the resolution of obesity-related comorbidities and mid-term weight loss rates between both techniques, Shoar and Saber have emphasized similar results in a meta-analysis of comparative studies, but RNYGB was favored with better weight loss rates in long-term follow-up.^25^ Although Bhandari *et al.* have shown a parallel increase in EWL percentages of LSG and RNYGB in the early postoperative period, RNYGB has come into prominence with better weight loss, weight loss maintenance, and resolution of type 2 diabetes.^26^ Melissas *et al.* have similarly noticed the superiority of RNYGB with better resolution rates for diabetes, hypertension, dyslipidemia, and sleep apnea in the first postoperative year.^27^ In addition, a nationwide cohort study from Sweden by Backman *et al.* have studied long-term effects of RNYGB procedure over pharmacological treatment and resolution rates of type 2 diabetes and have presented that gastric bypass surgery not only induces remission of pharmacological treatment of type 2 diabetes but also protects from the new onset of pharmacological diabetes treatment.^28^

In contrast to these findings, recent literature has mentioned both techniques as safe and efficient procedures in the management of obesity and related diseases. LSG has been described as a stand-alone procedure that preserves the intestine's normal anatomy and absorptive capacity with fewer risks of nutritional deficiencies.^29^ D'Hondt *et al.* have demonstrated better to excellent tolerance in 95.2% of patients in the LSG group with lower food tolerance rates than the nonobese patients who had no surgery.^30^ LSG as a single-stage procedure has been propagated in several studies regarding favored clinical outcomes such as enhanced quality of life, weight loss evolution, and resolution of type 2 diabetes, hypertension, and sleep apnea following short-/long-term after the surgery.^30–34^ Garg *et al.* have highlighted that both LSG and RNYGB have a similar impact on type 2 diabetes, hypertension, and obstructive sleep apnea, but RNYGB has better weight loss than LSG in the early 2-year postoperative period.^35^ Osland *et al.* have demonstrated in a meta-analysis of randomized controlled trials that both LSG and RNYGB procedures have exhibited comparable results regarding weight loss and the resolution of diabetic and nondiabetic comorbid diseases during the short-term follow-up.^5,36,37^ Buchwald *et al.* have mentioned 83% of the resolution rate of type2 diabetes with RNYGB in a meta-analyses over 22,000 patients.^38^ Boza *et al.* have presented similar resolution rates of type 2 diabetes for both LSG and RNYGB (87% vs. 91%, respectively).^39^ In our study, both LSG and RNYGB achieved a significant improvement in weight loss and obesity-related comorbidities. Resolution rates of type 2 diabetes, hypertension, and sleep apnea were 76%, 68%, and 100%, respectively, and these results were comparable with the literature.

Limitations to this study include small sample size and short follow-up interval owing to single-center experience at a rural hospital. Management of the pre- and postoperative care of obese patients should be established by a multidisciplinary team cautiously. Patient loss to follow-up and difficulties in the patients' multidisciplinary management in the rural setting are some drawbacks of this study. Approximately 20% of the patients were super-obese and presented with BMI >50 kg/m^2^. The LSG procedure was planned as an initial first-stage approach, where lifestyle modification and dietary regimen failure were observed. The RNYGB procedure was preferred in obese patients with severe comorbid diseases unable to maintain with regular prescriptions, including type 2 diabetes, hypertension, and other obesity-related diseases, and as a second-stage procedure in patients with EWL <40% on the sixth postoperative month following sleeve gastrectomy. However, none of the super-obese and obese patients in the LSG group required a second-stage gastric bypass procedure. Therefore, this study has the chance to compare the early results of the sleeve gastrectomy and gastric bypass procedure in terms of weight loss evolution, complication rates, and resolution of comorbidities.

In conclusion, LSG is a trending bariatric procedure with comparable clinical outcomes according to gastric bypass surgery. Early results of this study revealed that sleeve gastrectomy could be administered not only as an adjunctive but also as a sole approach in routine clinical practices to manage obesity-related risk factors during the early postoperative period. In this study, LSG has come into prominence with an easy learning curve, lower postoperative complication rates, and favorable outcomes regarding obesity-related comorbidities. Surgeons should also practice LSG safely and effectively in a rural setting. Further prospective studies and long-term results of this study should be performed with a larger cohort of patients.
